# Abscopal effect in a patient with chronic lymphocytic leukemia during radiation therapy: a case report

**DOI:** 10.1186/1757-1626-2-204

**Published:** 2009-11-18

**Authors:** Preethi Bangalore Lakshmanagowda, Lokesh Viswanath, Naveen Thimmaiah, Lokanath Dasappa, Sanjay Sudakar Supe, Pramod Kallur

**Affiliations:** 1 Department of Physiology, M.S. Ramaiah Medical College, Bangalore, India; 2 Department of Radiation Oncology, Kidwai Memorial Institute of Oncology, Bangalore, India; 3 Department of Medical Oncology, Kidwai Memorial Institute of Oncology, Bangalore, India; 4 Department of Radiation Physics, Kidwai Memorial Institute of Oncology, Bangalore, India

## Abstract

Radiotherapy has a significant impact on the local tumor environment and its distant component. Abscopal effect is the bystander effect of radiotherapy observed at a site distant to that irradiated within the same subject. Abscopal effect even though described, is not a common clinical event. We report a documented observation of abscopal effect in a patient of Chronic Lymphocytic Leukemia during radiation therapy.

## Introduction

The Abscopal effect is the effect of radiation therapy sometimes observed as response of tumour masses remote from the site of irradiation [1]. The term "abscopal effect" caused by radiotherapy was first coined by Mole in 1953 [2]. The word abscopal is derived from Latin *ab *means "position away from" and *scopos meaning *"a target for shooting at."

Much of the observed physiological Abscopal effect has been associated with splenic irradiaition[1]. Abscopal effect even though described, is not a common clinical event after radiotherapy, there have been reports of such effects in variety of malignancies including lymphoma, papillary adenocarcinoma, melanoma, adenocarcinoma of the esophagus, chronic lymphocytic leukemia, and hepatocellular carcinoma [3-10].

## Case presentation

A 65 year old female patient, a known case of Chronic Lymphocytic Leukemia since two years, presented to us with a massive right axillary lymphadenopathy with severe pain and neurovascular pressure effects in the axilla.

This patient at diagnosis, about two years back had presented with Right Axillay lymphadenopathy (approx 6-8 cms, Multiple) and was initially treated with chemotherapy COPP (cyclophosphamide, vincristine, procarbazine, prednisone) regimen for 3 cycles and then switched over to Chlorambucil & prednisone. Since she had disease progression in spite of active treatment all Chemotherapy drugs were stopped two months prior to presentation with us and symptomatic care with analgesics instituted.

On examination the patient had a massive right axillary lymphadenopathy, with multiple matted lymph nodes. The biggest node measured 14 × 12 × 10 cms with other axillary nodes ranging from 5 to 4 cms. She also had multiple (>1 cms) bilateral cervical lymphnodes (the largest 2 × 2 cms) at presentation were located in the right level II neck region away from the field of radiotherapy. She had no generalized lymphadenopathy or splenomegaly.

**Figure 1:**
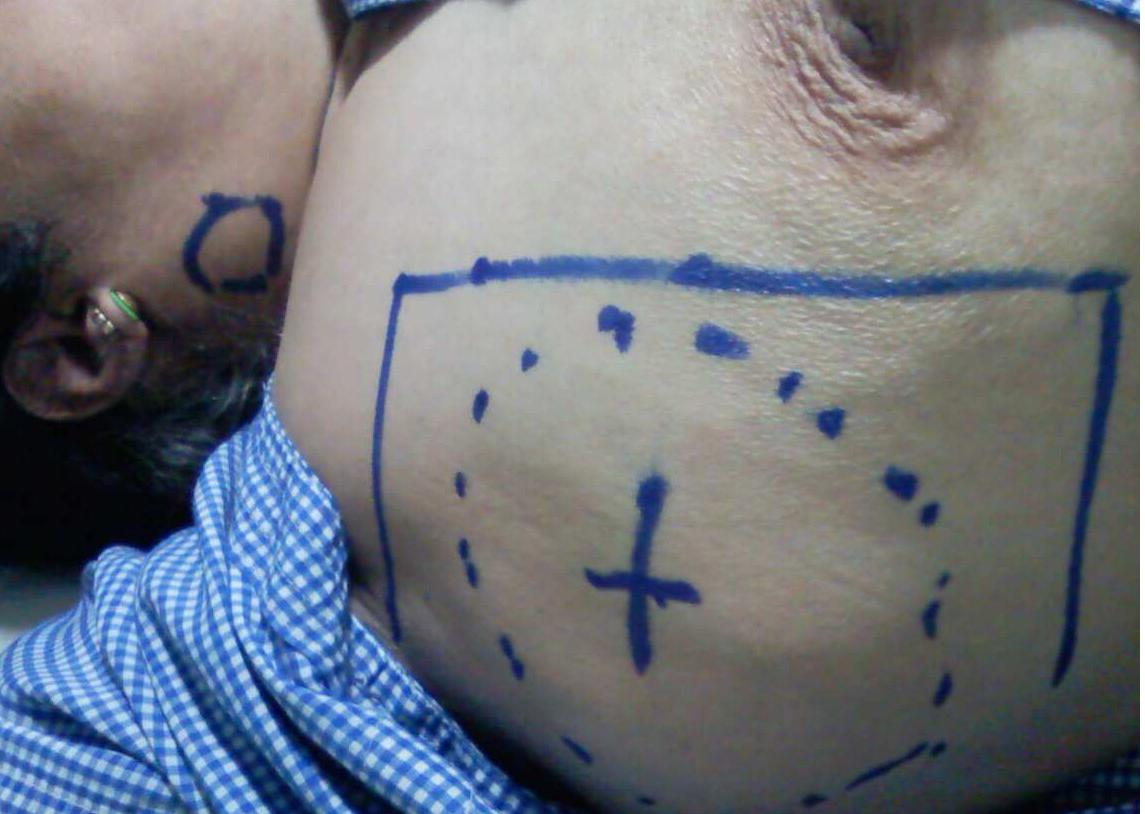
**Massive Axillary Lymphnode is marked in dotted line, the radiotherapy field is shaped like a rectangle and one of the multiple bilateral neck lymphnode (2 × 2 cms) away from the radiotherapy field is marked as small circle in the neck**.

The patient was treated with local field Radiation therapy to axilla with anterior & posterior parallel opposed local fields, to a dose of 2400 cGy in 12 fractions, 5 fractions per week (Figure 1).

**Figure 2:**
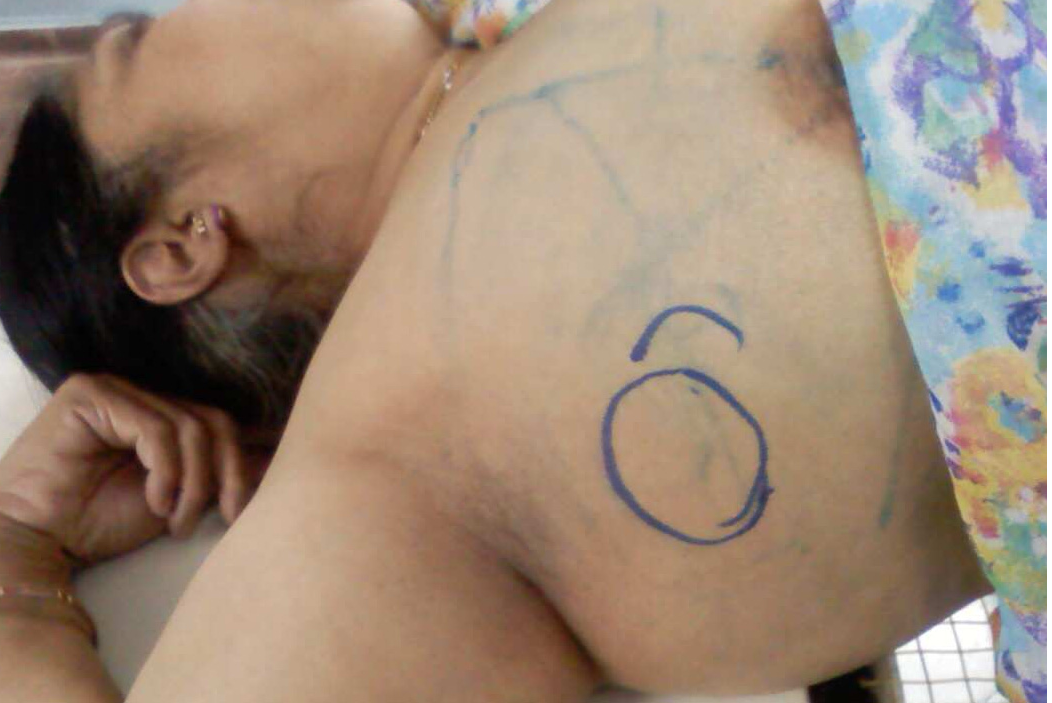
**the irradiatied axillary lymph node shows greater than 50% response at the end of radiotherapy and the unirridiated neck node away from the site of radiation has completely disappeared due to Abscopal effect**.

One week after radiotherapy the lymphnodes in the neck which was unirradiated, and away from the field of radiotherapy, started regressing and by the end of two weeks of radiotherapy the lymphnodes in the neck had shown complete regression due to abscopal effect and the axillary node had a partial response with subjective improvement in symptoms and performance status (Figure 2). 6 months after radiation therapy the patient continues to have sustained palliation in the irradiatied and distant site.

## Discussion & Conclusion

Radiotherapy has a significant impact on the local microenvironment of tissues within the radiation portal. Cells treated with Ionizing radiation sustain damage to its nuclear & cytoplasmic components, inducing of apoptosis, up-regulation transcription factors. Resulting in increased vascular permeability, altered cytokine levels and local inflammation [11,12].

Radiotherapy to one cell has direct impact on an adjacent cell resulting in bystander effect [13,14]. There have been two main theories proposed to explain the abscopal antitumor effect. The first applies to leukemias and lymphomas, it is hypothesized that during splenic irradiation diseased lymphocytes circulate through the irradiated volume (spleen), as the splenic size decreases the remotely located masses also decrease in size, giving an impression of a systemic antitumor effect from local treatment [1,4,5]. The second applies to solid tumors, it is postulated that local radiation induces a release of mitotic inhibitors (cytokines) into the circulation that mediate a systemic antitumor effect. It has been demonstrated that an elevation of circulating tumor necrosis factor after radiotherapy that coincided with the regression of a hepatocellular carcinoma situated away from the radiation field [2,10].

Others proposed hypothesis is that the abscopal effect is mediated by the immune system. Irradiation of tumour in one site induces release of circulating tumor antigen or inflammatory factors that may then mediate an augmented immune response against unirradiated, malignant lesions expressing similar tumor antigens. It has been shown that local radiotherapy increases the activity of natural killer cells [15,16].

In our patient by virtue of this unusual presentation, we were able to predict and observe the rare abscopal effect in the site distant from the site of irradiation. Irradiation of the disease in the axilla resulted in tumor mass regression in untreated distant site.

## Consent

A written informed consent was obtained from the patient and her son for publication of this case report and accompanying images. A copy of the written consent is available for review by the Editor-in-Chief of this journal. The patient has given their informed consent for this case report to be published. The editorial office may request copies of the consent documentation at any time.

## Competing interests

The authors declare that they have no competing interests.

## Authors' contributions

BLP analyzed and interpreted the patient data regarding the physiological effect in the patient & was the referring physician. VL & TN performed the examination & Radiotherapy treatment and DL is the medical oncologist treating the patient with Chemotherapy. BLP & VL are contributor in writing the manuscript. KPR and P helped in literature survey. SS has contributed to the Radiobiology interpretation of the Case report.
